# Associations between the Quality of Life and Nasal Polyp Size in Patients Suffering from Chronic Rhinosinusitis without Nasal Polyps, with Nasal Polyps or Aspirin-Exacerbated Respiratory Disease

**DOI:** 10.3390/jcm9040925

**Published:** 2020-03-28

**Authors:** Sven Schneider, Nicholas J. Campion, Sergio Villazala-Merino, David Tianxiang Liu, Tina Bartosik, Lukas D. Landegger, Navid Ahmadi, Christian A. Mueller, Erich Vyskocil, Victoria Stanek, Tamara Quint, Christine Bangert, Julia Eckl-Dorna

**Affiliations:** 1 Department of Otorhinolaryngology, Medical University of Vienna, 1090 Vienna, Austria; sven.schneider@meduniwien.ac.at (S.S.); nicholas.campion@meduniwien.ac.at (N.J.C.); sergio.villazalamerino@meduniwien.ac.at (S.V.-M.); david.liu@meduniwien.ac.at (D.T.L.); tina.bartosik@meduniwien.ac.at (T.B.); lukas.landegger@meduniwien.ac.at (L.D.L.); navid.ahmadi@meduniwien.ac.at (N.A.); christian.a.mueller@meduniwien.ac.at (C.A.M.); erich.vyskocil@meduniwien.ac.at (E.V.); victoria.stanek@meduniwien.ac.at (V.S.); 2 Department of Dermatology, Medical University of Vienna, 1090 Vienna, Austria; tamara.quint@meduniwien.ac.at (T.Q.); christine.bangert@meduniwien.ac.at (C.B.)

**Keywords:** nasal polyps, chronic rhinosinusitis, aspirin-exacerbated respiratory disease, Sino-Nasal Outcome Test-20 German Adapted Version

## Abstract

Chronic rhinosinusitis (CRS) is a common disease that substantially impairs the quality of life (QoL). Here, we aimed to assess patients’ QoL in different subtypes of CRS and correlated this with nasal polyp size to improve the clinical understanding of the burden of disease. In this retrospective single-center study, 107 patients with the following diagnoses were analyzed: CRS without nasal polyps (CRSsNP), CRS with nasal polyps (CRSwNP), or aspirin-exacerbated respiratory disease (AERD). Sino-Nasal Outcome Test-20 German Adapted Version (SNOT-20 GAV) scores and their correlation with endoscopic Total Polyp Scores (TPS) were evaluated. The mean SNOT-20 GAV scores were highest in patients with AERD (AERD = 43.4, CRSwNP = 36.3, CRSsNP = 30.9). A statistically significant correlation of total SNOT-20 GAV score with TPS was observed in CRSwNP patients (r = 0.3398, *p* = 0.0195), but not in AERD patients (r = 0.2341, *p* = 0.1407). When analyzing single SNOT-20 parameters, a strong correlation with TPS was observed for blockage/congestion of the nose, particularly in AERD patients (r = 0.65, *p* < 0.0001). The impact of nasal polyp size on the QoL differs amongst the subgroups of CRS. Nasal symptoms have the greatest impact on QoL in patients suffering from AERD. CRSwNP and AERD patients should be separately analyzed in clinical investigations and interpretations due to significant differences in QoL.

## 1. Introduction

Chronic Rhinosinusitis (CRS) is a common, yet complex and heterogeneous disease. Taking into account geographic variations, the reported prevalence of CRS ranges from 4% to 16% in North American and European countries [1,2,3,4,5]. CRS is classified as primary and secondary CRS. Primary disease is further stratified by anatomic distribution of disease into either localized (unilateral) or diffuse (bilateral) [5]. The phenotypes of CRS are attributed to underlying types of inflammation involving type 2 or non-type 2 patterns [5]. In contrast to CRSsNP (CRS without nasal polyps), CRSwNP (CRS with nasal polyps) is associated with the type 2 inflammation endotype. Between 2.7 and 4.4% of the population are affected by CRSwNP [5]. Within those with nasal polyps, approximately 10% suffer from aspirin-exacerbated respiratory disease (AERD), a syndrome that is characterized by adult-onset asthma and hypersensitivity to non-steroidal anti-inflammatory (NSAIDs) drugs, in addition to nasal polyposis [6]. Apart from causing sino-nasal symptoms, all of the subtypes of CRS also affect the sleep, mood, cognition, and productivity of patients. Thus, CRS not only represents a life quality impairing disease, but also an enormous socioeconomic burden that causes direct and indirect health costs of up to 21.4 billion $ per year in the US alone [1]. 

Different grading systems have been developed for objectively assessing disease severity and to help monitor therapeutic interventions. Objective measurements include the Lund–Mackay score, which quantifies the extent of disease in the paranasal sinuses by computed tomography (CT) as well as endoscopic scoring systems, such as the total polyp score (TPS) and Lund–Kennedy score [7,8]. Of those, the TPS is widely used in clinical studies, due to its ease of use and high reproducibility [9,10,11]. In addition, patient-reported outcome measures (PROMs) have become an important study tool. They do not only quantitatively assess patients’ symptom burden, but are also sensitive to the evaluation of clinical changes and thus, allow the monitoring of therapeutic success [12,13,14,15,16]. Of the PROMs that were developed for specifically addressing sino-nasal diseases, the SNOT-22 (Sino-Nasal Outcome Test) questionnaire and SNOT-20 German adapted version (GAV) are valid, reliable, and widely used instruments [12,14,15]. 

However, patients’ perception of their symptoms, as measured by PROMs, does not always correlate with objective measurements. In this respect, a poor association of total SNOT scores with CT-based Lund–Mackay scores or endoscopic based polyp grading scoring systems has been observed [16,17,18,19,20]. Only for the subcategory “nasal symptoms”, or for single parameters within this subcategory, have positive correlations with polyp size been observed in some [17,18,19,20], but not all [16], of the referenced studies. Of note, these studies assessed the CRSwNP population as a whole, without taking distinct subtypes of this disease into account. 

Although the clinical presentation of CRS subtypes might be similar, they have different triggers (e.g., fungal infection or aspirin sensitivity) and underlying pathophysiological mechanisms. Hence, the severity of symptoms as well as the impact on patient quality of life (QoL) may vary. In this respect, patients with non-type 2 disease or CRSsNP suffer from fewer recurrences of the disease after surgical treatment and have a lower incidence of comorbidities than seen in type-2 inflammation or CRSwNP [4]. Furthermore, patients with AERD have a higher endoscopic polyp score [21], greater impairment of olfactory function [22,23], and a higher risk of revision surgery when compared to those suffering from nasal polyposis alone [24,25]. However, despite clear differences in CRS subtypes, studies evaluating whether this is also reflected in QoL outcomes are missing. 

It is unknown whether impact of polyp extent varies in different types of CRS. Further evaluation of symptom patterns might help to distinguish and characterize patient groups. Therefore, the aim of this study was to investigate the association of patients’ subjective symptom burden measured by the SNOT-20 GAV with polyp size, as determined by the TPS scoring system in different subtypes of CRS. As this study was conducted at an academic tertiary health care center, all patients had severe CRS, often requiring surgery. This setting also allowed us to include a high number of patients suffering from AERD. 

## 2. Experimental Section

### 2.1. Study Population 

The Ethical Committee of the Medical University of Vienna approved the study (EK 1630/2019, 2 July 2019). Patients (aged 18 and older) presenting to the Department of Otorhinolaryngology and the Department of Dermatology at the Medical University of Vienna between 2017 and 2019 were included in this study. Patients who were diagnosed with vasculitis, cystic fibrosis, odontogenic sinusitis, fungal sinusitis, sarcoidosis, or autoimmune disease were excluded. 

Patients’ gender and age were recorded. An assessment of SNOT-20 GAV and nasal polyposis by endoscopy were performed during the same visit by a trained otorhinolaryngologist. The diagnosis of CRS was based upon the criteria according to the European Position Paper on Rhinosinusitis and Nasal Polyps (EPOS) 2020 [5]. Patients were stratified for the absence or presence of nasal polyps as well as the clinical diagnosis of AERD. Patients were specifically asked for the presence of the following symptoms by an ENT clinician: nasal blockage, congestion or stuffiness of the nose, nasal discharge or postnasal drip, facial pain or pressure, headache, and reduction/loss of smell. Furthermore, they were examined by nasal endoscopy for confirmation of the clinical diagnosis and the assessment of polyp presence and size [5]. Patients were classified as CRSwNP if nasal polyps were present at any time in the patients’ medical history. Hence, patients’ diagnosis remained CRSwNP or AERD if nasal polyps were not present at the time of the study visit due to recent surgical removal. In a subgroup analysis, we compared patients who had surgery without recurrence of polyps and thus a TPS = 0 to those who had a TPS of at least 1. Patients with TPS = 0 are marked “CRSwNP (TPS = 0) or AERD (TPS = 0)” in the respective results sections. The diagnosis of AERD was defined according to the European Academy of Allergy and Clinical Immunology (EAACI) position paper as the documented presence of nasal polyps, asthma, and respiratory symptoms upon the ingestion of aspirin or other non-steroidal anti-inflammatory drugs [26].

### 2.2. Outcome Measures

Nasal polyp size was graded by nasal endoscopy using the TPS system. Each side of the nasal cavity was separately evaluated and scored in a range from 0–4 (0 = no polyps, 1 = small polyps in the middle meatus not reaching below the inferior border of the middle turbinate, 2 = polyps reaching below the lower border of the middle turbinate, 3 = large polyps reaching the lower border of the inferior turbinate or polyps medial to the middle turbinate, and 4 = large polyps causing complete obstruction of the inferior nasal cavity) [9,11]. The sum of scores for both nasal cavities was recorded as the TPS value. 

QoL was recorded using the German version SNOT-20 GAV of the validated Sino-Nasal Outcome Test-20 (SNOT-20) [12,15]. Each parameter of the test is graded from 0–5 (0 = no problem, 1 = very mild problem, 2 = mild or slight problem, 3 = moderate problem, 4 = severe problem, and 5 = problem is as bad as it can be). Thus, the potential total SNOT-20 GAV score ranges from 0 to 100. The 20 questions were grouped in four subcategories: Nasal symptoms (“blockage/congestion of nose”, “sneezing”, “runny nose”, “post-nasal discharge”, “thick nasal discharge”, “need to clear throat/dry throat”, “cough”, “sense of smell”), otologic symptoms (“ear congestion”, “ear pain”, “dizziness”, “facial pain/pressure”), sleep symptoms (“difficulty falling asleep”, “waking up at night”, “fatigued or tired during the day“, “reduced productivity”, “reduced concentration”, “frustration, restlessness, irritability”), and emotional symptoms (“sad”, “embarrassed”). Each category, as well as each individual symptom, was analyzed. 

### 2.3. Statistical Analysis

The patient characteristics and TPS were described as mean (+/−standard deviation). One-way ANOVA was used to compare differences between groups as indicated in the respective figure legends (Graphpad Prism 7, Graphpad Software Inc., La Jolla, CA, USA). The Kruskal-Wallis test was employed for the subcategory analysis where we compared SNOT in groups with TPS of 0 to those with a TPS greater than 0 where Gaussian (normal) distribution could not be assumed due to the small sample size. Where applicable, pairwise group comparison was subsequently performed using Tukey’s multiple comparison test (for ANOVA) or Dunn’s multiple comparison test (for Kruskal-Wallis test). A *p*-value below 0.05 was considered to be significant. The correlation was calculated using Pearson’s correlation coefficient, and the significance levels are indicated. The strength of association was classified, as follows: weak r = (+/−)0.1–0.3, moderate r = (+/−)0.3–0.5, and strong r = (+/−)0.5–1. A *p*-value below ≤ 0.05 was considered as significant [27]. 

## 3. Results

### 3.1. Patient Characteristics

In this study, 122 patients presenting to the outpatient clinic of the Department of Otorhinolaryngology or the Department of Dermatology at the Medical University of Vienna were initially recruited. SNOT-20 GAV questionnaires of 15 patients were incomplete and had to be excluded, resulting in a total of 107 patients being analyzed. Of those, 43 (40%) were female and 64 (60%) were male. 19 patients (18%) were diagnosed with CRSsNP, 47 (44%) with CRSwNP and 41 (38%) with AERD (Table 1).

**Figure 1:**
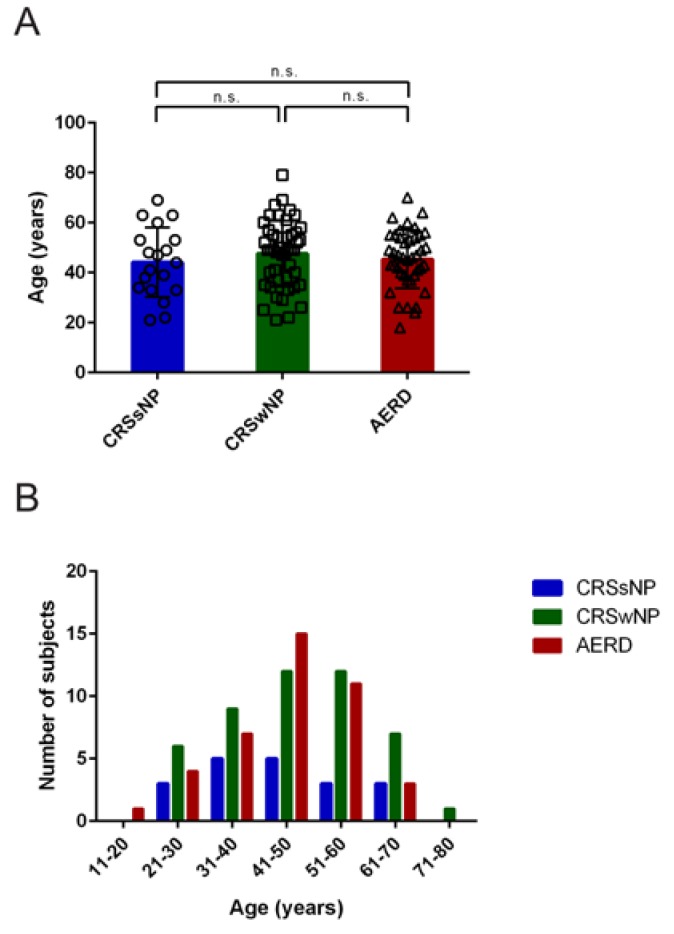
Age distribution in patients suffering from chronic rhinosinusitis without nasal polyps (CRSsNP, blue), chronic rhinosinusitis with nasal polyposis (CRSwNP, green) or chronic rhinosinusitis with polyps and aspirin-exacerbated respiratory disease (AERD, red). (**A**) Average age (y-axis, years) in the different patient groups (x-axis). No significant differences between groups were observed (one-way ANOVA *p* = 0.5391). Bars represent mean values with standard deviation (SD), values of individual patients are shown by single circles (CRSsNP), squares (CRSwNP), or triangles (AERD). (**B**) The number of subjects (y-axis) per age group (x-axis) is represented by bars in respective patient groups. n.s. = not significant.

The mean age of patients was 46.1 (Standard deviation (SD) = 12.7) years. There were no statistically significant differences that were observed between the mean ages of the three patient groups (Figure 1A). Patients suffering from nasal polyposis (with or without AERD) were predominantly in the age group that ranged from 41–60 years, whereas patients with chronic rhinosinusitis in the absence of polyposis were mainly between 31 and 50 years of age (Figure 1B).

Of the CRSsNP patients, 26% (*n* = 5) had had prior surgery as compared to 49% (*n* = 23) and 93% (*n* = 38) in the CRSwNP and AERD groups, respectively. The average number of prior surgeries was 1.2 (SD = 1.7), 1.3 (SD = 0.6) and 3.0 (SD = 2.7) in the three groups. Table 1 displays detailed information on patient characteristics.

### 3.2. Highest TPS and SNOT-20 GAV Scores were Observed in Patients suffering from AERD

The mean TPS was 3.41 in the CRSwNP and 4.02 in the AERD patient group (Table 2). The mean total SNOT-20 GAV score was 30.89 for patients with CRSsNP, 36.32 for patients suffering from CRSwNP, and 43.41 for the AERD group.

Significant differences in SNOT-20 GAV scores were only observed between patients suffering from AERD and CRSsNP (*p* = 0.0488). Upon the exclusion of patients who had recently undergone surgery without yet suffering from recurrence (CRSwNP *n* = 2, AERD *n* = 9) and, thus, had a TPS of 0 in the analysis, it became evident that AERD patients had significantly higher SNOT-20 GAV scores (ANOVA *p* = 0.0021) in comparison to CRSsNP (*p* = 0.0028) and CRSwNP patients (*p* = 0.0192). There was no statistically significant difference in SNOT-20 GAV scores between CRSsNP and CRSwNP (*p* = 0.4095) when patients with TPS = 0 (due to recent surgical treatment) were excluded from both groups.

### 3.3. AERD Patients Suffer More from Nasal Symptoms as Compared to CRSsNP and CRSwNP Patients

SNOT-20 GAV items were grouped into four thematic categories “nasal symptoms”, “otologic symptoms”, “sleep symptoms”, and “emotional symptoms”, as previously described [17,19]. No significant differences in SNOT-20 GAV score were observed between patient groups with regard to otologic, sleep, and emotional symptoms (ANOVA otologic *p* = 0.2295, sleep *p* = 0.6067, emotional *p* = 0.4317).

**Figure 2:**
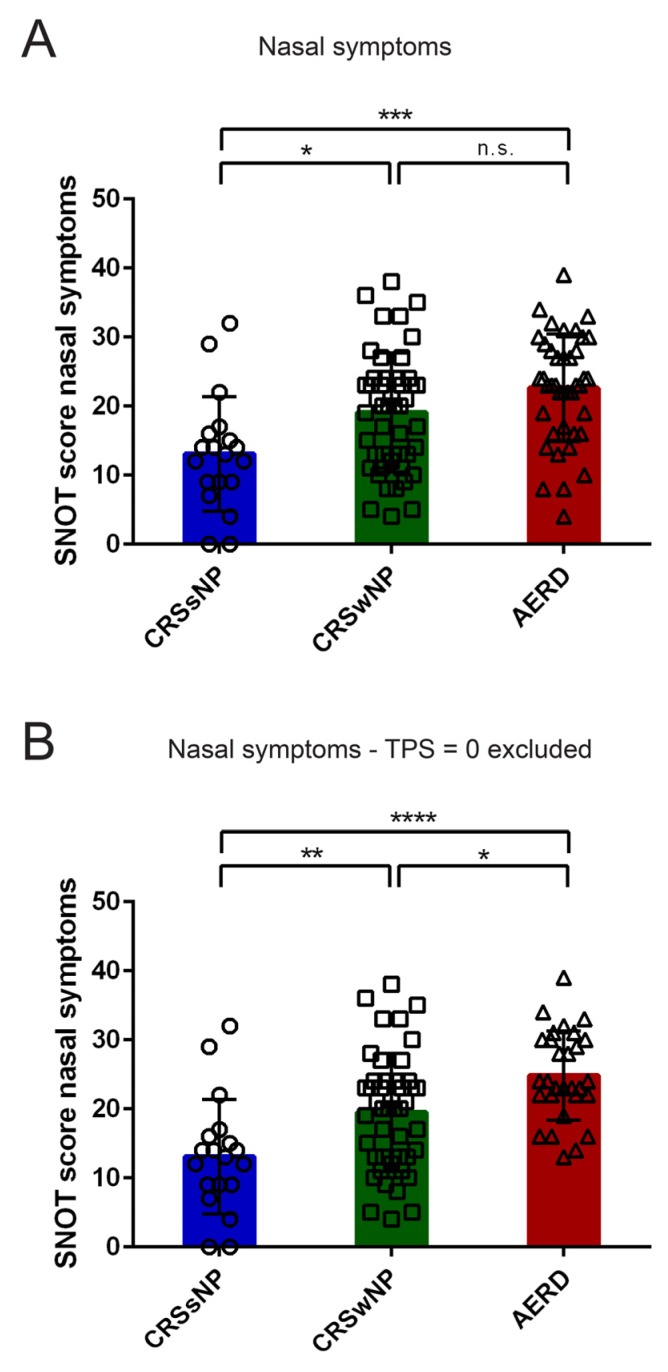
Subcategory analysis of Sino-Nasal Outcome Test-20 German Adapted Version (SNOT-20 GAV) score in patients suffering from chronic rhinosinusitis without nasal polyps (CRSsNP, blue), chronic rhinosinusitis with nasal polyposis (CRSwNP, green), or chronic rhinosinusitis with nasal polyps and aspirin-exacerbated respiratory disease (AERD, red). Score for (**A, B**) Nasal symptoms in (**A**) all patients and (**B**) upon the exclusion of patients with documented polyp disease but TPS = 0 (excluded: CRSwNP *n* = 2; AERD *n* = 9) are shown in the respective patient groups (x-axis). Bars represent mean values with standard deviation; values of individual patients are depicted as circles (CRSsNP), squares (CRSwNP), or triangles (AERD). Significant differences between patient groups were observed (One-way ANOVA (**A**) *p* = 0.0003, (**B**) *p* ≤ 0.0001), significant differences in pairwise comparisons using Tukey’s multiple comparison test are indicated (*: *p* ≤ 0.05; **: *p* ≤ 0.01; ***: *p* ≤ 0.001; ****: *p* ≤ 0.0001, n.s. = not significant).

However, the highest SNOT-20 GAV scores were observed in AERD patients with regard to nasal symptoms, which differed significantly from CRSsNP in the total patient population (Figure 2A) and from the CRSwNP group if patients with documented nasal polyps with TPS = 0 were excluded (Figure 2B).

### 3.4. Quality of Life in AERD and CRSwNP Patients after Surgery in a Subset of Patients

**Figure 3:**
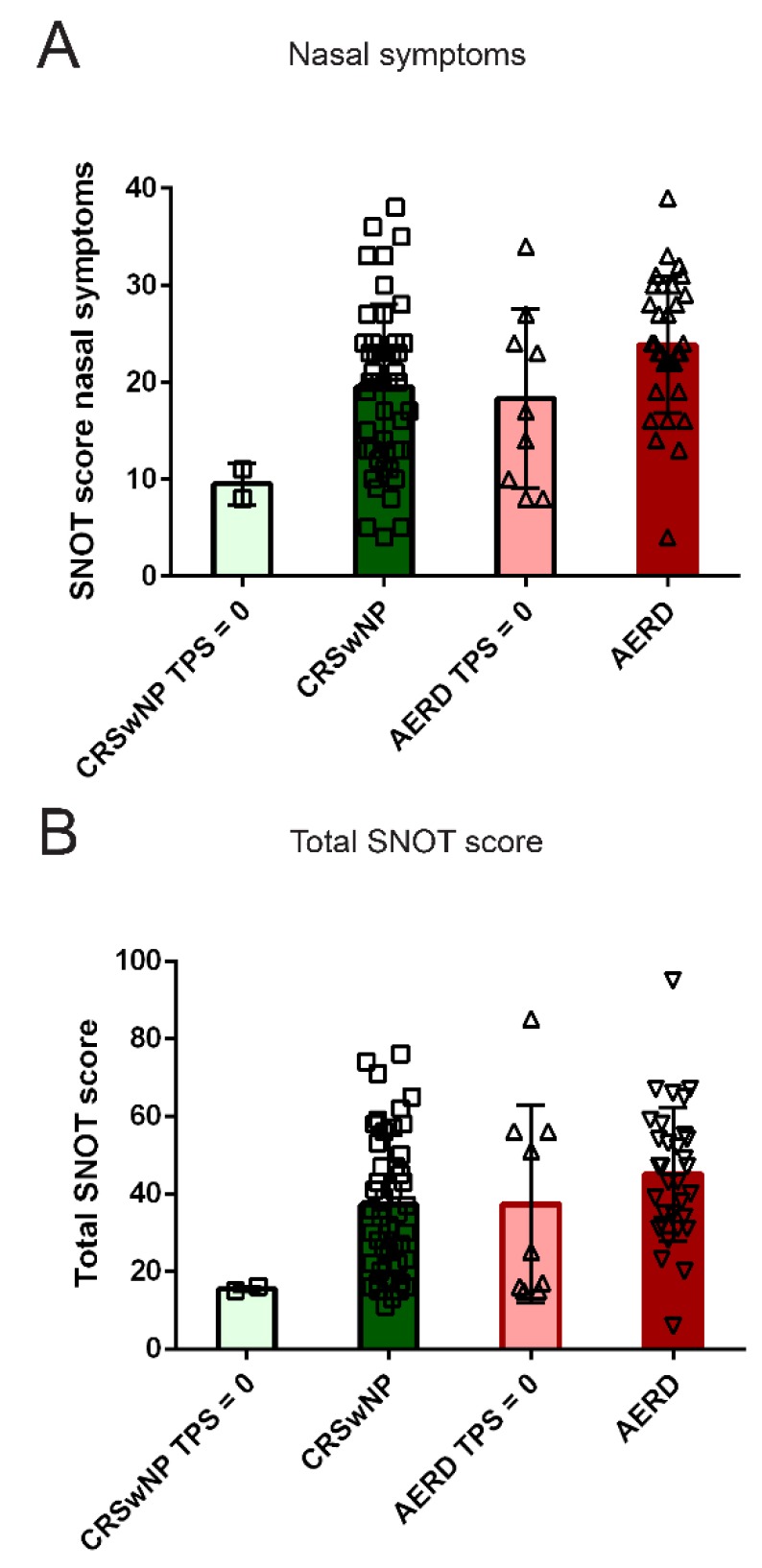
Subcategory analysis of Sino-Nasal Outcome Test-20 German Adapted Version (SNOT-20 GAV) score in patients suffering from chronic rhinosinusitis with nasal polyposis (CRSwNP, green) or chronic rhinosinusitis with nasal polyps and aspirin-exacerbated respiratory disease (AERD, red) with a total nasal polyp score (TPS) greater than 0 (dark colors) or a TPS of 0 (light colors) (TPS = 0: CRSwNP *n* = 2; AERD *n* = 9). Score for (**A**) Nasal symptoms and (**B**) Total SNOT score (all y-axes) are shown in the respective patient groups (x-axis). Bars represent mean values with standard deviation; values of individual patients are depicted as squares (CRSwNP) or triangles (AERD). Kruskal–Wallis test indicated significant differences between patient groups in total and nasal symptom Sino-Nasal Outcome Test-20 (SNOT) score ((**A**) *p* = 0.0181 and (**B**) *p* = 0.0396). However, no significant differences in pairwise comparisons using Dunn’s multiple comparison test were observed.

Next, we addressed the impact of surgical treatment on the QoL by comparing the values of SNOT-20 GAV in polyp patients with recent surgery without current polyp recurrence and, thus, TPS of 0 and in patients with a TPS > 0 in the CRSwNP and AERD group. AERD patients’ QoL, as measured by different SNOT categories, was comparable in subjects with current polyps and subjects with a TPS = 0 (Figure 3 and data not shown).

CRSwNP patients whose polyps had been removed by surgery showed lower total and nasal symptom subcategory SNOT scores. However, the *post-hoc* tests revealed no significant differences amongst the groups.

### 3.5. Correlation of TPS with Nasal Symptoms in Patients with AERD but not with CRSwNP

The correlation of TPS with total SNOT-20 GAV scores, subcategory scores, as well as single item scores was performed for CRSwNP and AERD patients. When the analysis was performed in the combined group of AERD patients and the CRSwNP patients’ cohort, total SNOT-20 GAV scores were significantly, but weakly, correlated with TPS (r = 0.29; *p* = 0.0056) (Table 3).

Stratified analysis showed significant moderate correlation in the CRSwNP group (r = 0.3398, *p* = 0.0195), but not in AERD patients (r = 0.2341, *p* = 0.1407).

Furthermore, subcategory analyses of “Nasal symptoms” revealed a moderate association with TPS in the whole cohort of patients with nasal polyps (r = 0.32; *p* = 0.0024), as well as in AERD patients alone (r = 0.37, *p* = 0.016), but not in patients suffering from CRSwNP (r = 0.24, *p* = 0.1063). A single item (“Blockage/Congestion of nose”) was the only symptom, which robustly correlated with TPS in both groups (whole cohort: r = 0.53; *p* < 0.0001, CRSwNP: r = 0.40; *p* = 0.0051, AERD: r = 0.65; *p* < 0.0001). The items “Sneezing” (r = 0.31; *p* = 0.0462), “Runny nose” (r = 0.35; *p* = 0.0279), and “Sense of smell” (r = 0.42; *p* = 0.0066) were significantly correlated to TPS in AERD patients, but not in CRSwNP patients. 

Otologic symptoms showed no correlation in any of the investigated groups, aside from “Dizziness”, which correlated with the TPS in the CRSwNP group (r = 0.31; *p* = 0.0361).

In the subcategory “Sleep symptoms”, a significant moderate correlation with TPS was observed for the CRSwNP group (r = 0.32; *p* = 0.0289), but not for AERD patients. The elements “Difficulty falling asleep” (r = 0.32; *p* = 0.0280) and “Reduced productivity” (r = 0.30; *p* = 0.0392) correlated significantly with moderate association with TPS values in the CRSwNP group. Furthermore, in this group, the subcategory “Emotional symptoms” (r = 0.33; *p* = 0.0246) as well as the single item “Sad” (r = 0.29; *p* = 0.0470) was significantly correlated with TPS values.

## 4. Discussion

Chronic rhinosinusitis is a heterogeneous disease, comprising various pathophysiological processes that can have a significant impact on the QoL. The association of impaired QoL with disease subtypes and polyp size has so far been poorly investigated and therefore represented the main aim of this study. This is the first study including a large cohort of patients suffering from AERD (*n* = 41). In summary, we found that amongst the investigated CRS subtypes, AERD patients appear to suffer from the most burdensome symptoms, particularly nasal symptoms.

In this study, we observed that AERD patients have significantly higher SNOT-20 GAV scores when compared to CRSwNP or CRSsNP. This is in accordance with previous data showing that QoL and olfactory function of AERD patients is more compromised when compared to those CRS patients who are not aspirin-intolerant [21,23]. In our analysis of the various subcategories of the SNOT—nasal, otologic, sleep, and emotional symptoms—it became evident that the different SNOT outcome is mainly due to nasal symptom burden. In contrast to other studies, our CRSsNP population did not score worse than the CRSwNP patients with regard to fatigue or sleep [28] or facial pain [29]. One potential explanation for this could be the selection of our study cohort. Being based at an academic tertiary health care center, all of the patients included had pronounced CRS requiring surgical intervention in the past or near future. The potential selection bias is one limitation of our study. This also impacted on our sub-analysis where patients who underwent polyp surgery and had a TPS = 0 were analyzed. CRSsNP patients do less frequently require additional appointments at our tertiary health care center and they are further treated elsewhere. Thus, we did not have a control group of CRSsNP patients having undergone recent surgery and no signs of CRS and some of the analyses may have been underpowered due to the retrospective nature of the study. 

We aimed to evaluate the association of polyp size with QoL in different CRS entities. Recent findings correlating QoL with objective measurements of nasal burden show a poor correlation between the two [16,17,18,19,20]. For example, in postoperative patients, neither CT-scores evaluated by Lund–Mackay score nor endoscopic findings that were assessed by Lund–Kennedy score correlated with SNOT-20 values [18]. In our study, we observed significantly higher impact on QoL in patients presenting with nasal polyps compared to CRSsNP patients. Although this finding might also be influenced by underlying inflammatory mechanisms and not exclusively by polyp extent, severe impact on QoL can be expected when nasal polyps are present. However, the subtypes of CRSwNP are rarely analyzed separately in the literature. Data from our study suggest that it is important to take both polyp size and the underlying pathogenesis into account when interpreting SNOT values. Our findings that the SNOT-20 GAV subcategory “nasal symptoms” only correlates with TPS in AERD patients, whilst polyp size was associated with “sleep” and “emotional symptoms” in CRSwNP patients (see Table 3) indicates that polyps in these groups might cause different symptoms and suffering. Thus, the interpretation of patient outcomes in clinical studies would benefit from separate analysis of TPS and SNOT-20 by the CRS groups. 

We performed a sub-analysis, where we compared patients with CRSwNP or AERD with a recent history of polyp surgery to those without recent surgical intervention. Although only being conducted in a small group of patients, it revealed an interesting observation. Graphical representation indicated that CRSwNP patients with recent surgery scored lower in total and all SNOT-20 GAV subcategories. These results indicate that the QoL in AERD patients might only be improved by surgery slightly and/or for a short period of time. Limited olfactory function [23] and a higher rate of polyp recurrence still hampered postsurgical AERD patients. Whilst CRSwNP patients show a polyp recurrence rate of 40% within 40 months after surgery, 58% of AERD patients suffer from polyp regrowth in the same time span [24,25]. Therefore, maximum intensified treatment should be considered for AERD patients, regardless of the absence or presence of nasal polyps. 

Besides surgery, AERD might be treated by aspirin desensitization, thereby simultaneously reducing polyp size, nasal, and asthmatic symptoms [30,31,32,33,34], as well as improving olfactory function [22]. However, the life-long intake of aspirin is associated with severe side effects, causing a high number of patients to discontinue the treatment [31,32]. Lately, biologicals targeting interleukin 4 (IL-4), IL-5, IL-13, or immunoglobulin E (IgE) have shown promising effects in patients suffering from CRSwNP [34]. Whether these agents are equally effective in patients with AERD remains to be determined.

## 5. Conclusions

This is the first study assessing the differences in QoL between CRSwNP and a large cohort of AERD patients in relation to the extent of polyposis. The aim of our study was to evaluate patients with pronounced CRS, typical for a tertiary health care center. We found that, although symptoms of CRS can be overlapping, significant differences in QoL in the evaluated subtypes of CRS can be observed. Our findings suggest that SNOT-20 GAV values differ in the subgroups of CRS and the extent of nasal polyposis might have a variable impact on QoL in CRSwNP or AERD patients. The results of this study might help to characterize QoL patterns in different phenotypes of CRS. Hence, in future clinical studies, it would be advisable to additionally stratify and analyze CRS patients by underlying pathology according to inflammation type and AERD, separately, rather than by the presence or absence of polyps alone.

## Figures and Tables

**Table 1:** Patient characteristics.

		CRSsNP (*n* = 19)	CRSwNP (*n* = 47)	AERD (*n* = 41)
Gender (m/f)		15/4	31/16	18/23
Age				
	mean	44.11	47.62	45.41
	range	21–69	21–79	18–70
Total polyp score				
	mean	0.00	3.40	4.02
	range	0	0–8	0–8
Total SNOT score				
	mean	30.89	36.31	43.41
	range	4–73	11–76	6–95
History of prior surgery (y/n)		5/14	23/24	38/3

CRSsNP = Chronic rhinosinusitis without nasal polyposis, CRSwNP = Chronic rhinosinusitis with nasal polyposis, AERD = Aspirin-exacerbated respiratory disease, m = male, f = female, SNOT = Sino-Nasal Outcome Test-20 German Adapted Version, y/n = yes/no.

**Table 2:** Total Polyp and SNOT-20 GAV score in patients suffering from chronic rhinosinusitis without nasal polyps (CRSsNP), chronic rhinosinusitis with nasal polyposis (CRSwNP), or chronic rhinosinusitis with nasal polyps and aspirin-exacerbated respiratory disease (AERD).

	Average Value ± Standard Deviation			*p*-Value of Pairwise Comparison		
	CRSsNP	CRSwNP	AERD	CRSsNP-CRSwNP	CRSsNP-AERD	CRSwNP-AERD
**Total polyp score**	0 ± 0	3.40 ± 2.01	4.02 ± 2.89	**<0.0001**	**<0.0001**	0.3985
**Total SNOT-20 GAV score**	30.89 ± 19.92	36.32 ± 18.17	43.41 ± 19.23	0.5434	**0.0488**	0.1891
**Total SNOT-20 GAV score if patients with TPS = 0 excluded**	N/A	37.24 ± 18.01	49.14 ± 17.03		**0.0028**	**0.0192**

Total polyp score (TPS) and total Sino-Nasal Outcome Test-20 German Adapted Version (SNOT-20 GAV 20) scores (in all patients or selectively in patients with a TPS greater than 0) are shown in the respective patient groups. Significant differences between patient groups were observed, as follows: (One-way ANOVA TPS: *p* < 0.0001, total SNOT-20 GAV score *p* = 0.0443, total SNOT-20 GAV score if patients with TPS = 0 excluded: *p* = 0.0021). *p*-values of differences in pairwise comparisons using Tukey’s multiple comparison test are indicated. N/A = Not available. SD, Standard deviation. Bold indicated significant *p*-values.

**Table 3:** Individual correlations of SNOT-20 GAV scores of subcategories with total nasal polyp score.

		All Patients		CRSwNP		AERD	
		r	*p*-value	r	*p*-value	r	*p*-value
Total SNOT score		**0.29**	**0.0056**	**0.34**	**0.0195**	0.23	0.1407
Nasal symptoms		**0.32**	**0.0024**	0.24	0.1063	**0.37**	**0.0160**
	Need to clear throat/dry throat	0.06	0.6112	0.22	0.1431	−0.10	0.5424
	Sneezing	**0.27**	**0.0108**	0.16	0.2905	**0.31**	**0.0462**
	Runny nose	**0.22**	**0.0423**	0.06	0.6753	**0.35**	**0.0279**
	Cough	0.06	0.5892	0.09	0.5571	−0.01	0.9689
	Post-nasal discharge	0.10	0.3560	0.02	0.9063	0.14	0.3793
	Thick nasal discharge	0.20	0.0627	0.20	0.1916	0.18	0.2631
	Sense of smell	**0.33**	**0.0017**	0.25	0.0974	**0.42**	**0.0066**
	Blockage/Congestion of nose	**0.53**	**<0.0001**	**0.40**	**0.0051**	**0.65**	**<0.0001**
Otological symptoms		0.11	0.3038	0.21	0.1551	0.01	0.9412
	Ear congestion	0.10	0.3405	0.04	0.7751	0.11	0.4821
	Dizziness	0.09	0.3917	**0.31**	**0.0361**	−0.08	0.6082
	Ear pain	0.07	0.5225	0.12	0.4170	0.03	0.8549
	Facial pain/pressure	0.06	0.5882	0.17	0.2444	−0.05	0.7478
Sleep symptoms		**0.22**	**0.0374**	**0.32**	**0.0289**	0.13	0.4143
	Difficulty falling asleep	0.20	0.0548	**0.32**	**0.0280**	0.14	0.3932
	Waking up at night	0.14	0.1814	0.23	0.1234	0.10	0.5436
	Fatigued or tired during the day	**0.21**	**0.0465**	0.28	0.0600	0.12	0.4448
	Reduced productivity	**0.22**	**0.0360**	**0.30**	**0.0392**	0.14	0.3974
	Reduced concentration	0.20	0.0694	0.25	0.0958	0.15	0.3474
	Frustration, restlessness, irritability	0.08	0.4867	0.15	0.3283	0.04	0.8043
Emotional symptoms		**0.27**	**0.0106**	**0.33**	**0.0246**	0.21	0.1835
	Sad	**0.23**	**0.0299**	**0.29**	**0.0470**	0.16	0.3105
	Embarrassed	0.21	0.0516	0.25	0.0938	0.18	0.2679

Displayed are Pearson’s correlation coefficient and significance levels (*p*-value). Significant correlations are bold.

## References

[1] DeConde A.S., Soler Z.M. (2016). Chronic rhinosinusitis: Epidemiology and burden of disease. Am. J. Rhinol. Allergy.

[2] Hastan D., Fokkens W.J., Bachert C., Newson R.B., Bislimovska J., Bockelbrink A., Bousquet P.J., Brozek G., Bruno A., Dahlen S.E. (2011). Chronic rhinosinusitis in europe—An underestimated disease. A ga(2)len study. Allergy.

[3] Chen Y., Dales R., Lin M. (2003). The epidemiology of chronic rhinosinusitis in canadians. Laryngoscope.

[4] Bachert C., Akdis C.A. (2016). Phenotypes and emerging endotypes of chronic rhinosinusitis. J. Allergy Clin. Immunol. Pract..

[5] Fokkens W.J., Lund V.J., Hopkins C., Hellings P.W., Kern R., Reltsma S., Toppila-Salmi S., Bernal-Sprekelsen M., Mullol J., Alobid I. (2020). European position paper on rhinosinusitis and nasal polyps 2020. Rhinology.

[6] Rajan J.P., Wineinger N.E., Stevenson D.D., White A.A. (2015). Prevalence of aspirin-exacerbated respiratory disease among asthmatic patients: A meta-analysis of the literature. J. Allergy Clin. Immunol..

[7] Ting F., Hopkins C. (2018). Outcome measures in chronic rhinosinusitis. Curr. Otorhinolaryngol. Rep..

[8] Psaltis A.J., Li G., Vaezeafshar R., Cho K.S., Hwang P.H. (2014). Modification of the lund-kennedy endoscopic scoring system improves its reliability and correlation with patient-reported outcome measures. Laryngoscope.

[9] Meltzer E.O., Hamilos D.L., Hadley J.A., Lanza D.C., Marple B.F., Nicklas R.A., Adinoff A.D., Bachert C., Borish L., Chinchilli V.M. (2006). Rhinosinusitis: Developing guidance for clinical trials. J. Allergy Clin. Immunol..

[10] Bachert C., Mannent L., Naclerio R.M., Mullol J., Ferguson B.J., Gevaert P., Hellings P., Jiao L., Wang L., Evans R.R. (2016). Effect of subcutaneous dupilumab on nasal polyp burden in patients with chronic sinusitis and nasal polyposis: A randomized clinical trial. JAMA.

[11] Gevaert P., Calus L., Van Zele T., Blomme K., De Ruyck N., Bauters W., Hellings P., Brusselle G., De Bacquer D., van Cauwenberge P. (2013). Omalizumab is effective in allergic and nonallergic patients with nasal polyps and asthma. J. Allergy Clin. Immunol..

[12] Piccirillo J.F., Merritt M.G., Richards M.L. (2002). Psychometric and clinimetric validity of the 20-item sino-nasal outcome test (snot-20). Otolaryngol. Head Neck Surg..

[13] Gray S.T., Phillips K.M., Hoehle L.P., Caradonna D.S., Sedaghat A.R. (2017). The 22-item sino-nasal outcome test accurately reflects patient-reported control of chronic rhinosinusitis symptomatology. Int. Forum Allergy Rhinol..

[14] Morley A.D., Sharp H.R. (2006). A review of sinonasal outcome scoring systems—Which is best?. Clin. Otolaryngol..

[15] Baumann I., Plinkert P.K., De Maddalena H. (2008). Development of a grading scale for the sino-nasal outcome test-20 german adapted version (snot-20 gav). HNO.

[16] Hopkins C., Browne J.P., Slack R., Lund V., Brown P. (2007). The lund-mackay staging system for chronic rhinosinusitis: How is it used and what does it predict?. Otolaryngol. Head Neck Surg..

[17] Dejaco D., Riedl D., Huber A., Moschen R., Giotakis A.I., Bektic-Tadic L., Steinbichler T., Kahler P., Riechelmann H. (2019). The snot-22 factorial structure in european patients with chronic rhinosinusitis: New clinical insights. Eur. Arch. Otorhinolaryngol..

[18] Ryan W.R., Ramachandra T., Hwang P.H. (2011). Correlations between symptoms, nasal endoscopy, and in-office computed tomography in post-surgical chronic rhinosinusitis patients. Laryngoscope.

[19] Sedaghat A.R., Gray S.T., Caradonna S.D., Caradonna D.S. (2015). Clustering of chronic rhinosinusitis symptomatology reveals novel associations with objective clinical and demographic characteristics. Am. J. Rhinol. Allergy.

[20] DeConde A.S., Bodner T.E., Mace J.C., Alt J.A., Rudmik L., Smith T.L. (2016). Development of a clinically relevant endoscopic grading system for chronic rhinosinusitis using canonical correlation analysis. Int. Forum Allergy Rhinol..

[21] Jang D.W., Comer B.T., Lachanas V.A., Kountakis S.E. (2014). Aspirin sensitivity does not compromise quality-of-life outcomes in patients with samter’s triad. Laryngoscope.

[22] Gudziol V., Michel M., Sonnefeld C., Koschel D., Hummel T. (2017). Olfaction and sinonasal symptoms in patients with CRSwNP and AERD and without AERD: A cross-sectional and longitudinal study. Eur. Arch. Otorhinolaryngol..

[23] Katotomichelakis M., Riga M., Davris S., Tripsianis G., Simopoulou M., Nikolettos N., Simopoulos K., Danielides V. (2009). Allergic rhinitis and aspirin-exacerbated respiratory disease as predictors of the olfactory outcome after endoscopic sinus surgery. Am. J. Rhinol. Allergy.

[24] DeConde A.S., Mace J.C., Levy J.M., Rudmik L., Alt J.A., Smith T.L. (2017). Prevalence of polyp recurrence after endoscopic sinus surgery for chronic rhinosinusitis with nasal polyposis. Laryngoscope.

[25] Morrissey D.K., Bassiouni A., Psaltis A.J., Naidoo Y., Wormald P.J. (2016). Outcomes of modified endoscopic Lothrop in aspirin-exacerbated respiratory disease with nasal polyposis. Int. Forum Allergy Rhinol..

[26] Kowalski M.L., Agache I., Bavbek S., Bakirtas A., Blanca M., Bochenek G., Bonini M., Heffler E., Klimek L., Laidlaw T.M. (2019). Diagnosis and management of nsaid-exacerbated respiratory disease (n-erd)-a eaaci position paper. Allergy.

[27] Cohen J. (2012). Statistical Power Analysis for the Behavioral Sciences.

[28] Greguric T., Trkulja V., Baudoin T., Grgic M., Smigovec I., Kalogjera L. (2016). Differences in the sino-nasal outcome test 22 and visual analog scale symptom scores in chronic rhinosinusitis with and without nasal polyps. Am. J. Rhinol. Allergy.

[29] Dietz de Loos D.A., Hopkins C., Fokkens W.J. (2013). Symptoms in chronic rhinosinusitis with and without nasal polyps. Laryngoscope.

[30] Sweet J.M., Stevenson D.D., Simon R.A., Mathison D.A. (1990). Long-term effects of aspirin desensitization--treatment for aspirin-sensitive rhinosinusitis-asthma. J. Allergy Clin. Immunol..

[31] Berges-Gimeno M.P., Simon R.A., Stevenson D.D. (2003). Long-term treatment with aspirin desensitization in asthmatic patients with aspirin-exacerbated respiratory disease. J. Allergy Clin. Immunol..

[32] Cho K.S., Soudry E., Psaltis A.J., Nadeau K.C., McGhee S.A., Nayak J.V., Hwang P.H. (2014). Long-term sinonasal outcomes of aspirin desensitization in aspirin exacerbated respiratory disease. Otolaryngol. Head Neck Surg..

[33] Cooper T., Greig S.R., Zhang H., Seemann R., Wright E.D., Vliagoftis H., Cote D.W.J. (2019). Objective and subjective sinonasal and pulmonary outcomes in aspirin desensitization therapy: A prospective cohort study. Auris Nasus Larynx.

[34] Ren L., Zhang N., Zhang L., Bachert C. (2019). Biologics for the treatment of chronic rhinosinusitis with nasal polyps-state of the art. World Allergy Organ. J..

